# Supplementary material for: COZOID: contact zone identifier for visual analysis of protein-protein interactions
Source: BMC Bioinformatics. 2018 Apr 6;19:125. doi: 10.1186/s12859-018-2113-6 (PMC5889581; doi:10.1186/s12859-018-2113-6)
Supplement: Supplementary file 2 — Software build. Executable binary file of the software tool. (ZIP 118,784 kb) [file 12859_2018_2113_MOESM2_ESM.zip › cozoid/java/docs/beansbinding-1.2.1-doc.zip!/org/jdesktop/el/impl/parser/ParseException.html]

ParseException (Beans Binding)


---


|  |  |  |
| --- | --- | --- |
| **Overview** \| **Package** \| **Class** \| **Use** \| **Tree** \| **Deprecated** \| **Index** \| **Help** | |  |
| **PREV CLASS**   **NEXT CLASS** | **FRAMES**    **NO FRAMES**     **All Classes** |  |
| SUMMARY: NESTED \| FIELD \| CONSTR \| METHOD | DETAIL: FIELD \| CONSTR \| METHOD |  |


---


## org.jdesktop.el.impl.parser Class ParseException

```
java.lang.Object
  java.lang.Throwable
      java.lang.Exception
          org.jdesktop.el.impl.parser.ParseException
```

**All Implemented Interfaces:**: java.io.Serializable

---

``` public class ParseException extends java.lang.Exception ```

This exception is thrown when parse errors are encountered.
You can explicitly create objects of this exception type by
calling the method generateParseException in the generated
parser.
You can modify this class to customize your error reporting
mechanisms so long as you retain the public fields.

**See Also:**: Serialized Form

---

| **Field Summary** | |
| --- | --- |
| `Token` | `currentToken`             This is the last token that has been consumed successfully. |
| `protected  java.lang.String` | `eol`             The end of line string for this machine. |
| `int[][]` | `expectedTokenSequences`             Each entry in this array is an array of integers. |
| `protected  boolean` | `specialConstructor`             This variable determines which constructor was used to create this object and thereby affects the semantics of the "getMessage" method (see below). |
| `java.lang.String[]` | `tokenImage`             This is a reference to the "tokenImage" array of the generated parser within which the parse error occurred. |


**Constructor Summary**

`ParseException()`
  
          The following constructors are for use by you for whatever
purpose you can think of.

`ParseException(java.lang.String message)`

`ParseException(Token currentTokenVal,
int[][] expectedTokenSequencesVal,
java.lang.String[] tokenImageVal)`
  
          This constructor is used by the method "generateParseException"
in the generated parser.


| **Method Summary** | |
| --- | --- |
| `protected  java.lang.String` | `add_escapes(java.lang.String str)`             Used to convert raw characters to their escaped version when these raw version cannot be used as part of an ASCII string literal. |
| `java.lang.String` | `getMessage()`             This method has the standard behavior when this object has been created using the standard constructors. |

**Methods inherited from class java.lang.Throwable**

`fillInStackTrace, getCause, getLocalizedMessage, getStackTrace, initCause, printStackTrace, printStackTrace, printStackTrace, setStackTrace, toString`

**Methods inherited from class java.lang.Object**

`clone, equals, finalize, getClass, hashCode, notify, notifyAll, wait, wait, wait`

**Field Detail**

### specialConstructor

```
protected boolean specialConstructor
```

:   This variable determines which constructor was used to create
    this object and thereby affects the semantics of the
    "getMessage" method (see below).

---


### currentToken

```
public Token currentToken
```

:   This is the last token that has been consumed successfully. If
    this object has been created due to a parse error, the token
    followng this token will (therefore) be the first error token.

---


### expectedTokenSequences

```
public int[][] expectedTokenSequences
```

:   Each entry in this array is an array of integers. Each array
    of integers represents a sequence of tokens (by their ordinal
    values) that is expected at this point of the parse.

---


### tokenImage

```
public java.lang.String[] tokenImage
```

:   This is a reference to the "tokenImage" array of the generated
    parser within which the parse error occurred. This array is
    defined in the generated ...Constants interface.

---


### eol

```
protected java.lang.String eol
```

:   The end of line string for this machine.


**Constructor Detail**

### ParseException

```
public ParseException(Token currentTokenVal,
                      int[][] expectedTokenSequencesVal,
                      java.lang.String[] tokenImageVal)
```

:   This constructor is used by the method "generateParseException"
    in the generated parser. Calling this constructor generates
    a new object of this type with the fields "currentToken",
    "expectedTokenSequences", and "tokenImage" set. The boolean
    flag "specialConstructor" is also set to true to indicate that
    this constructor was used to create this object.
    This constructor calls its super class with the empty string
    to force the "toString" method of parent class "Throwable" to
    print the error message in the form:
    ParseException:

---


### ParseException

```
public ParseException()
```

:   The following constructors are for use by you for whatever
    purpose you can think of. Constructing the exception in this
    manner makes the exception behave in the normal way - i.e., as
    documented in the class "Throwable". The fields "errorToken",
    "expectedTokenSequences", and "tokenImage" do not contain
    relevant information. The JavaCC generated code does not use
    these constructors.

---


### ParseException

```
public ParseException(java.lang.String message)
```


**Method Detail**

### getMessage

```
public java.lang.String getMessage()
```

:   This method has the standard behavior when this object has been
    created using the standard constructors. Otherwise, it uses
    "currentToken" and "expectedTokenSequences" to generate a parse
    error message and returns it. If this object has been created
    due to a parse error, and you do not catch it (it gets thrown
    from the parser), then this method is called during the printing
    of the final stack trace, and hence the correct error message
    gets displayed.

    :   **Overrides:**: `getMessage` in class `java.lang.Throwable`

---


### add\_escapes

```
protected java.lang.String add_escapes(java.lang.String str)
```

:   Used to convert raw characters to their escaped version
    when these raw version cannot be used as part of an ASCII
    string literal.


---


|  |  |  |
| --- | --- | --- |
| **Overview** \| **Package** \| **Class** \| **Use** \| **Tree** \| **Deprecated** \| **Index** \| **Help** | |  |
| **PREV CLASS**   **NEXT CLASS** | **FRAMES**    **NO FRAMES**     **All Classes** |  |
| SUMMARY: NESTED \| FIELD \| CONSTR \| METHOD | DETAIL: FIELD \| CONSTR \| METHOD |  |


---
